# Indents

**Published:** 1906-07-15

**Authors:** 


### INDENTS.

**☞ Brief Articles of Technical or Scientific Value will receive notice and comment. Contributions invited.**

**To Prevent Bubbling of Borax.** Mix a minimum of gum-arabic and water with the borax on a glass slab.—*International Dental Journal.*

**Separating Varnish.** Dissolve three ounces of Borax in a quart of water, bring to the boiling degree and add six ounces of powdered shellac. A. Doughaday, in *Western Journal.*

**False Sympathy.** Countryman to the dentist, "Doctor, the tooth next to that one aches too." "Yes," replied the dentist, "it aches in sympathy." "Yank it out, too, Doc., durn such sympathy."

**Penalty for Accident.** A dentist of Paris, France, was indicted for causing his patient great suffering and injury and fined heavily, besides being sent to prison for one month. It seems that he gave the patient an anesthetic and extracted a number of teeth, and one of the roots fell back into the throat and was carried into the bronchus, where it caused bronchial pneumonia. It was finally expelled during a fit of coughing.

**Anesthetizing the Pulp without using the Pressure Syringe.** Prepare the cavity in the usual way. Place a saturated pledget of cotton with cocain solution directly over the pulp and fill the remainder of the cavity with a piece of vulcanite. Then take a short piece of orangewood, fit it into the cavity as prepared and direct the patient to bite down upon this with increasing force. In this way we can obtain a well-directed regulated force or pressure and with less discomfort to the patient and operator.

In our daily clinics we have succeeded in anesthetizing pulps by this method, after repeated attempts by other means have failed.—E. T. Loeffler, in *Summary*.

**Liquid Flux.** The best liquid flux that I have ever used, which I compound myself and find it ever ready and faithful is:

| | | |
|---|---|---|
| R | Boracic acid | |
| | Borax aa | ℥ ss. |
| | Ammonia muriate | grs. xii. |
| | Pot. carb | grs v. |
| | Aqua dist., q. s. | ℥ iv |

It will keep indefinitely and can be used on any metal for hard soldering. It should not be used, however, in connection with porcelain.—H. E. Davis, in *Dentist's Magazine*.

**Adrenalin Chlorid as an Adjunct to Cocain in Local Anesthesia.** I have used the combination of cocain and adrenalin in varying proportions and I find that the following prescription produces universally good results.

| | |
|---|---|
| Cocain (P. D. & Co's hypodermatic tablet No. 510) | 1-6 grain. |
| Adrenalin Chlorid Solution | 1 drop. |

Distilled extract witch hazel sufficient to fill syringe.
Inject as any local anesthetic.

When more cocain is used the Adrenalin chloride should be correspondingly increased. It is hardly necessary to advise my readers of the importance of sterilized needles, an aseptic field and the contra-indication for hypodermatic injections in close proximity to pus formations. If these be made, infection of tissue with pyogenic bacteria may result, and we may then hear another report of cocain causing suppuration.—Dr. W. C. Davis, Lincoln Nebraska.

**Treating an Alveolar Abscess.** I believe it is a mistake to carry a tooth along through weeks and weeks of treatment through the pulp chamber, and many of us make a mistake in treating teeth too often. Whenever an abscess forms about a root it nearly always surrounds the apex of the root. It destroys the peridental membrane around the sides of the root, consequently treatment through the root canal does not get at what we want and after pus has been present for some time, if the surface of the root has been bathed in pus for many weeks, we will get satisfactory results from treatment through the pulp chamber in very few cases. I do not practice immediate root filling in such cases, but I have done it "more immediately" than is the general custom. In such cases I prefer to fill the root canal, and, if necessary, make an opening through the alveolar process.—Arthur D. Black, Chicago, in *Review*.

**Method of Making Amalgam Dies.** Amalgam dies have become very popular for construction gold and porcelain inlays copes for crowns and for building up teeth that have decayed far below the gum margins, with gold.

Take an impression with pink base plate gutta percha in the following manner: Moisten the tooth and cavity with glycerine. Soften the gutta percha over an alcohol flame, being careful not to blister it, and force the gutta percha into the cavity with a broad instrument. The glycerine will prevent the gutta percha from sticking in the cavity. Flow a stream of cold water over the gutta percha to harden it; carefully remove and if the margins are perfect mix a small quantity of plaster, build it to a pyramid on a glass slab and invest the impression by forcing it down in the plaster with an instrument, being careful to have the plaster well up around the margins. When the plaster is hard mix the amalgam. I prefer to use alloy in the form of shavings or copper amalgam is better still, as it can be used over again several times by warming it until the mercury appears in the surface and then grind it thoroughly in a mortar. Carefully pack the amalgam around the impression until it is well covered and then roll up a piece of unvulcanized rubber about the size of a thimble, and wrap several thicknesses of rubber tape around this. Place it over the amalgam insert in a vise and squeeze out the mercury. Leave it under pressure three or four hours or over night. Then break away the plaster, remove gutta-percha and you will find a clean cut and polished die which is easily mounted in the swage and makes a perfect matrix.—C. J. Hadley, Chicago.

**Infected Third Molar Sockets.** The following treatment is suggested: First of all, the use of water—tepid water—sterilized, or, if preferred, the addition of boric acid. After washing thoroughly, the socket may require curettement to insure the removal of any abnormal growth resulting from the infection and the inability of the tissues to heal normally. In cases of continued pain, camphorated oil gives relief in mild cases, but if extreme anodynes are necessary, one-eighth grain morphia, incorporated with balsam Peru and iodoform (odorless), made into a paste and carried to the floor of the socket in a pledget of cotton and sealed with Fletcher's carbolized resin and cotton, reducing the size of the dressing as the socket closes. In case of enlarged sub-maxillary and parotid glands, the use of one part tincture of opium and two parts of lead water form a happy (external) application for the relief of pain, applied on flannel and wound with a hot cloth.—Jas. W. Winn, *Head Light.*

**Ingredients of the Dentifrice.** Powdered pumice stone: This, chemically, is a silicate of aluminum. It results as a light porous, hard stone, from the action of water vapor upon melted lava. It is added to dentifrices in the proportion of from 1 to 15 parts in the total 100 parts of dentifrice; it should be rarely employed in larger amount than 3 percent.

The following formula is of a dentifrice very largely used throughout Germany. It contains an unusually large proportion of pumice and it is to be used but two or three times a week, on other occasions a less abrasive dentifirice serving.

Take of

| | |
|---|---|
| Powdered soap | 1 drachm. |
| Borax | 1 " |
| Tannin | 1 " |
| Pumice | 3 drachms. |
| Orris Root | 4 " |
| Precipitated chalk | 1 ounce. |
| Oil of wintergreen | 15 minims. |

Mix.

When it is thought advisable to employ an additional abrasive, in a dentifrice composition, particularly if it be of so positive an action as pumice, care should be taken to prevent its indiscriminate use by those liable to be harmed by it, and in no case should it be employed for constant daily use. The cleansing effect of orange wood, and similar substances, largely rests, after eliminating the personal equation, upon the abrasive function performed by the silica always present in the wood fibre cell.

Chlorate of Potassium: We mention this substance first because popularly regarded as an oxidizing addition to dentifrices, washes, pastes, etc., it can under no conditions present in the human subject yield free oxygen. It is eliminated unchanged, principally through saliva and renal excretion.

Hobart Hare, in the Standard Dispensatory, describes it as being, after the cyanide, the most poisonous salt of potassium.

Its continued use, even in small amounts, would be an active cause of stomach derangement, and would unquestionably seriously affect patients suffering from any form of kidney disease.

The particular value of potassium chlorate is found in its antiseptic and germicidal action. It is a stimulant, in fact an irritant to mucous membranes, markedly stimulating the flow of saliva, and is unquestionably of service in various forms of stomatitis.

Prof. P. G. Unna, of Hamburg, strongly commends a paste composed of :—

| | |
|---|---|
| Precipitated chalk | 1 part. |
| Florentine oris root | 1 " |
| Glycerine and aromatics to make a paste. | |
| Add potassium chlorate powdered | 2 parts. |

To summarize the advantages accruing from an addition of chlorate of potassium to paste or dentifrice:—"Used in large amount (50 percent of the mix) it acts as a deodorant, stimulant, disinfectant and bactericide, hardens the gums, increases the flow of saliva." (Frederick F. Pound, D.D.S.)

The disadvantages, in the writer's opinion, are of such serious nature as to overbalance the undeniable good properties possessed by the substance, and the writer takes the position that chlorate of potassium is too harmful an agent to form an ingredient of any dentifrice, paste, or similar composition used indiscriminately without professional supervision.—Dr. H. H. Boom, in *Stomatologist.*

